# Humor and the willingness to buy healthy food posted on Instagram

**DOI:** 10.3389/fpsyg.2024.1419648

**Published:** 2024-08-13

**Authors:** Ester Reijnen, Lea Laasner Vogt, Daniele Catarci, Jean L. Zengaffinen, Sabine M. Bremermann-Reiser, Lars Bläuer

**Affiliations:** School of Applied Psychology, ZHAW Zurich University of Applied Sciences, Zurich, Switzerland

**Keywords:** healthy food, social media, purchase intention, position, joke content

## Abstract

Humorous messages (not derogatory jokes) related to obesity seem to be retweeted frequently. Potentially, such humor could be included in viral public health campaigns to combat obesity, but would jokes actually increase the likelihood of purchasing healthy foods advertised on social media? 411 participants were asked to test two soon-to-be-introduced features (e.g., repost button) in Instagram on their phones. Participants scrolled through a series of posts about various topics ranging from architecture to beauty products. A healthy food post, preceded by one of four *joke types* (two containing the word “fat”), was embedded at the top, middle or bottom position of the Instagram page. After participants scrolled through the page, perhaps reposting some posts, they were presented with the healthy food product featured in the post and were asked to indicate a purchase probability (0–100), as well as whether they recognized the food product from the post (yes/no). At the end of the study, the individual jokes were rated as “funny/not funny” and “positive/negative”. If the food product was recognized, the joke type played a role. In particular, jokes containing the word “fat” had a negative effect on the purchase probability. However, if the food product was not recognized, only its position on the page mattered. The purchase probability was highest if the product was placed at the top. Social media, criticized for promoting unhealthy food consumption, especially among children, can therefore also be used to address issues such as the global obesity epidemic.

## 1 Introduction

*“Dear food, either stop being delicious or stop making me fat.”*

A joke that may make you *laugh*, but does it also lead you subsequently to *buy healthier foods*, for example, in a grocery store or any other place where food is sold? Why might this question be important? Overweight (including obesity) has become one of the world’s biggest challenges (Jaacks et al., 2019; World Health Organization, 2020; Mohajan and Mohajan, 2023). This because it carries health consequences such as cardiovascular diseases (e.g., angina pectoris), diabetes, and some types of cancer which not only reduce life expectancy, but also the quality of life of those affected (Mohajan and Mohajan, 2023). Additionally, these obesity-related consequences result in enormous healthcare costs (Okunogbe et al., 2021; Ling et al., 2023). Yet, to date interventions that could tackle this challenge or crisis successfully are lacking.

Now it is known that people struggling with overweight regularly consult *social media* platforms (e.g., Facebook, Instagram, etc.) for advice on how to eat healthier and thereby lose weight (Chung et al., 2017). However, the growing popularity of these platforms has also prompted *food companies* to advertise their products on these platforms (Bragg et al., 2021). Unfortunately, the products promoted—also the ones by highly followed celebrities—are mostly unhealthy (see Freeman et al., 2014; Facebook; Dunlop et al., 2016; Potvin Kent et al., 2019; Reagan et al., 2020; Turnwald et al., 2022) and children in particular, tend to consume these advertised foods (Boyland et al., 2016). Hence, in social media there appears to be competition between the commercial voices of the “weight loss” and the “obesity promoting” industry, which, according to Dunlop et al. (2016) can only be solved by “creative *content* and resources” (p. 41). Note that this claim does not include the banning, for example, of unhealthy food advertisements, as this would have little effect anyhow (Sturm and Cohen, 2009; for fast food restaurants, but see Hingle and Kunkel, 2012; for children). More importantly, bans in general are also the least accepted interventions compared to, for example, labels, taxes, etc. (see Reynolds et al., 2019). Yet, what might that creative content be? Could it be humor? Or more specifically, humor linked in some way or the other to the content of an advertised product (e.g., Snicker’s “Diva” commercial). Giving humor a try makes sense, since humor is considered as one of the most appealing characteristics of advertisements (see Förster and Brantner, 2016).

Even though humor has been *used* extensively, especially in *commercial advertising* (TV, print; [e.g., Madden and Weinberger, 1984; Weinberger and Gulas, 1992]) empirical evidence^1^ regarding the effects of humor (ads seen as funny) on *purchase intention* of the advertised product is not only thin but mixed at best (see Eisend’s, 2009 meta-analysis, or Sternthal and Craig, 1973, for an overview; see also Sutherland, 2008). Furthermore, only a few (and mostly older) studies investigated how humor works. One of them is, for example, Gelb and Pickett’s (1983) study which found that *perceived humor* (here a joke about smoking with/without a satirical component) positively influenced the *liking of the ad*. Furthermore, the more the ad was liked, the more people indicated that they would purchase the advertised self-help QUIT-KIT, which is supposed to help quit smoking. The study did, however, not find a direct link of perceived humor on purchase intentions (see also Strick et al., 2009 for a similar result). Similarly, Bartos and Dunn (1976) argue that humor’s effect is primarily on ad liking. In other words, “funny” ads do not necessarily need to be *effective*. Hence, one should rather create ads that are liked, rather than ones that are humorous but less liked (see, however, Cadwell, 1981, who in contrast states that the mere perception or presence of humor is what makes advertising liked).

Furthermore, we know that attention is a necessary requisite to process visual objects to the point of being *recognizabl*e, for example, as a Snicker (e.g., Grill-Spector and Kanwisher, 2005 or Cohen et al., 2012) and hence also as an object for *purchase* (e.g., Nguyen et al., 2020). However, attention is a limited capacity, and as a result, 95% of our decisions—including food decisions—are made without attention. It is therefore important to know how attention influences the above-mentioned *relationship between ad liking and purchase intention*. For example, while Goodrich (2011) assumes that the relationship is most stable when attention is involved, Auty and Lewis (2004) assume the opposite. It is also unclear what role humor might play in this story. Given the lack of research in this area, we look at how emotional valences behave. In this regard, Nguyen et al. (2020) found that for decisions made with attention, the happier people were, the less they bought (here: unhealthy snacks); that is, their emotional valence was negatively associated with purchase. The opposite pattern was found for decisions made without attention. Based on these results we assume that humor or a joke may have a differential effect depending on whether attention is at play or not.

Similarly, Petty et al. (1983) state that message argument strength matters only if attention is used (or at play). That is, the presence of attention allows for the processing of the content of humorous ads or jokes, and hence, to produce differential effects.

On the other hand, the content of the joke should not matter if attention is not at play, since the content is not processed. In this case, we assume that only position effects would play a role. Thereby we draw on the literature on nudging—an approach developed in opposition to the understanding of humans as rational actors—which assumes that when people decide without attention (or unconsciously), their decisions are influenced by environmental (here: context) changes. For example, merely positioning snacks such as Snickers at a supermarket checkout counter increases the rate of their purchase (e.g., Piacentini et al., 2000; Thornton et al., 2012, see also Wansink and Hanks, 2013 or Reijnen et al., 2019). Hence, as we focus on the use of humor on social media (here: Instagram feeds) to test whether viral health campaigns can be used to combat overweight, examining position effects is key.

In summary: to our knowledge, there is no research about the use of humor on digital media, especially in relation to food. Furthermore, given the work by Bragg et al. (2021) showing that traditional ads (e.g., print, online banner) are perceived differently (e.g., likability, artistic appeal) than Instagram ads, the findings about humor in print media are probably only conditionally valid in social media. We therefore try to close the research gap regarding the effect of humor in social media on the purchase (intention) of healthy foods. We thereby used an artificial Instagram feed wherein we placed (in different positions) jokes of different content (here: with or without the word fat) in front of a healthy product being advertised. To evaluate the differential effects of joke content and position as a function of attention, we recorded whether the product was processed with/without attention.

## 2 Method

### 2.1 Participants

The 411 participants of this smartphone-based online study were students of the ZHAW Zurich University of Applied Sciences and were recruited via the university’s internal student e-mail list. The participants’ age ranged from 18 to 50 years (*M*_age_ = 24.19; *SD*_age_ = 4.24) and 70.6% of them were female. As compensation for participation, participants could enter a draw for an iPad (which 80.5% of all participants did) or, if a student of the School of Applied Psychology, receive course credit (which 10.9% of all participants did). All participants gave informed consent, and the study was conducted in accordance with the Declaration of Helsinki and approved by the Ethics Committee of the Canton of Zurich (protocol code Req-2024-00439; date of approval: April 9, 2024).

### 2.2 Stimulus material

**Figure 1:**
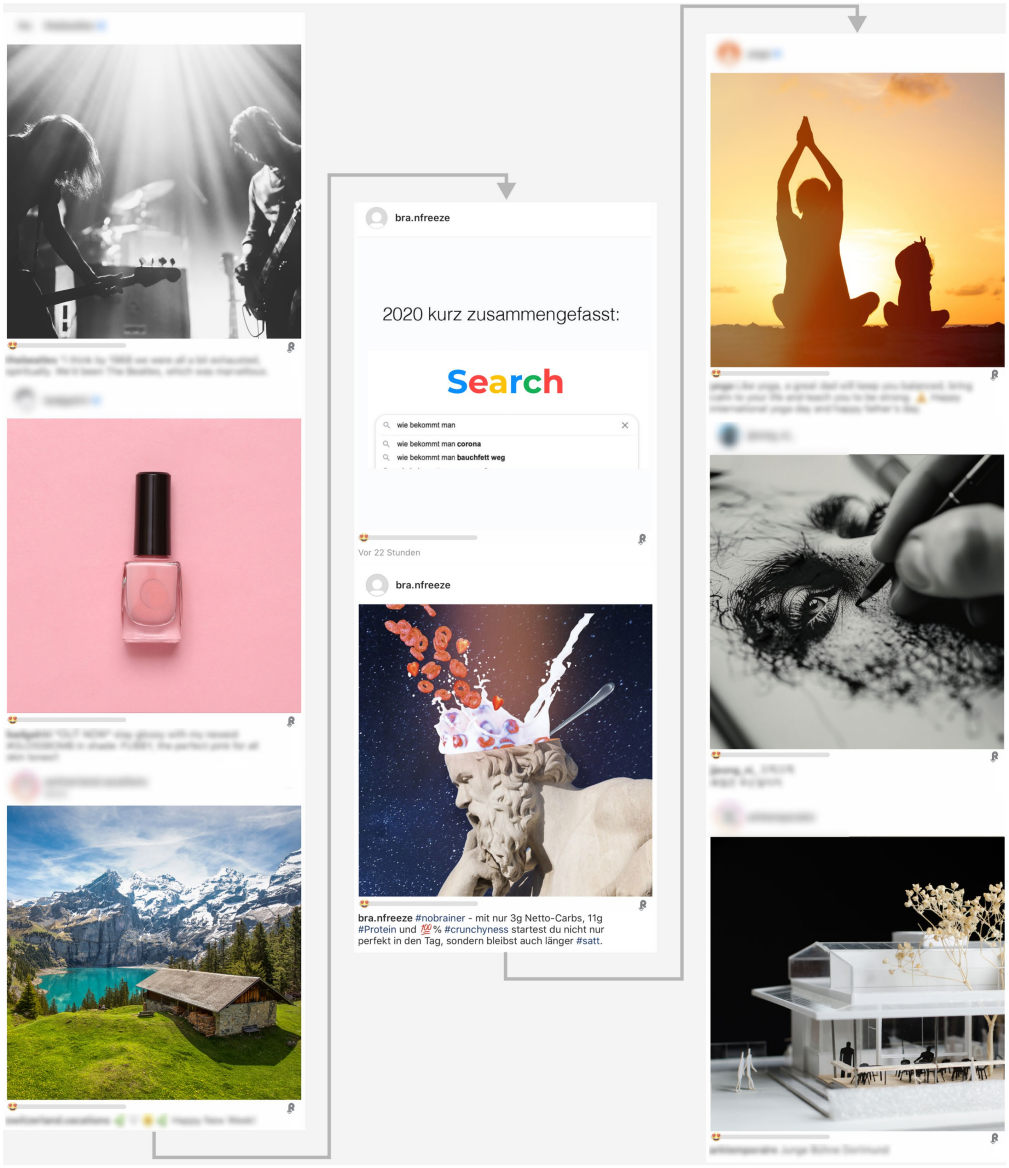
Artificial Instagram page. This is an example with the joke and the food-related post positioned in the middle. For copyright reasons, the original “filler” images have been replaced with similar licensed images from Adobe Stock and texts in the posts have been blurred. The image in the “food” post is the original, which has been modified from licensed images from Adobe Stock.

The stimulus material consisted of an overweight-related joke embedded in an artificial Instagram feed, displayed alongside a post about a healthy food and six filler posts (see Figure 1). The joke could be one of four possible joke types (named: Google, career, shape, and elevator). Two of the jokes (Google and career) explicitly included the word “fat” (the footnote^2^ contains the wording of the jokes in the original language and translations that attempt to convey the jokes’ intended humor and meaning). The joke could be positioned at the top, middle, or bottom of the page, but was always immediately followed by the critical healthy food post (strawberry cereals). The six so-called filler posts, which—depending on the position of the joke and the healthy food—were placed in varying numbers (0 vs. 6, 3 vs. 3, 6 vs. 0) before or after, covered topics ranging from architecture to beauty products. Each post (including the joke) contained a repost button and an emoji like slider.

### 2.3 Procedure and design

**Figure 2:**
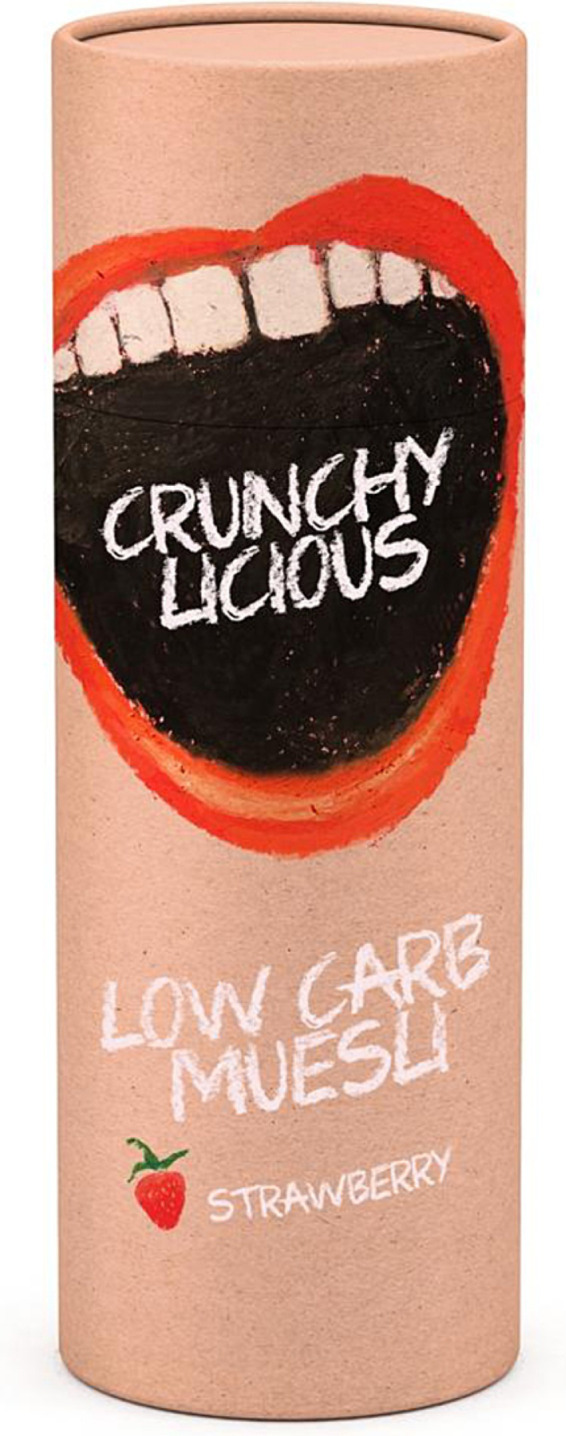
Healthy food product. The image is the original, which has been modified from licensed images from Adobe Stock.

At the beginning^3^ of the study (the online software *Unipark*, www.unipark.de, was used for presentation and data collection), participants were told via a cover story that they were to test two new Instagram features (the *repost button* and the *emoji like slider*—in reality, however, we tested the effect of the joke on the willingness to buy the healthy food presented in the subsequent post). Subsequently, participants were able to scroll through the page that contained the joke, the healthy food post as well as the filler posts. They could choose to click on the repost button or move the emoji like slider (range: 0 to 10—where the “10” stands for the maximum liking). Once they had scrolled through the page and clicked the “continue” button, they were presented with the healthy food product featured in the post. The participants were asked to indicate the probability of a purchase (0–100; see Figure 2), as well as whether they had recognized the food product from the post (yes/no). Thereafter they were asked to classify the joke regarding “funny/not funny” (funniness; a kind of arousal) and “positive/negative” (valence). Before their demographic data were assessed at the end of the study, they were asked some questions about their social-media behavior (e.g., which platforms they use). The resulting data setup is a 4 (joke type: Google, career, shape, and elevator) × 3 (position: top, middle, bottom) design; whereby participants were randomly assigned to one of the 12 conditions (i.e., one of the factor level combinations).

## 3 Results

In total, 11 participants (2.6%) who needed less than 180 or more than 2,700 s to complete the study were excluded from the analysis. Data was analyzed using IBM SPSS Statistics.

### 3.1 Repost button and emoji like slider

While the *repost* button was rarely used, the *emoji like slider* was (see Table 1). Regarding the likings (joke, healthy food post, mean of filler posts), they all differed^4^ significantly from each other (all *t*’s > 3.47, all *p*’s < 0.001; *t*-tests with Bonferroni correction); whereby the filler posts were liked most, followed by the joke. The least liked one was the healthy food post.

**Table 1:** Descriptive data.

			Post			
	Joke		Healthy food		Filler	
	*N*	*M (SD)*	*N*	*M (SD)*	*N*	*M (SD)*
Repost button	23	–	8	–	124	–
Emoji like slider						
Actively moved only	249	4.9 (3.8)	158	3.8 (3.3)	1,551	5.9 (2.2)
Not moved (= 0 incl.)	411	2.9 (3.8)	411	1.5 (2.8)	2,466	3.6 (2.2)

Each participant received 6 filler posts, hence the possible maximum or the total number is: 2,466. Under “only actively moved”, the values of participants who did not actively move the slider were excluded because it was not clear whether 0 represented their true value or whether they simply could not be bothered to move the slider. The pattern looks the same, there is only an upward/downward shift of the values.

Furthermore, there was a significant correlation between the liking of the healthy food post and its purchase intention, *r*(*N* = 411) = 0.30, *p* < 0.001; that is, the more the healthy food post is liked, the more likely the intention to purchase the food. More important, however, is the question of whether jokes can influence purchase intent, and if so, how (respectively to which of its attributes)?

### 3.2 Funniness (arousal) and valence

Depending on how participants classified the joke, they were assigned to one of the four conditions (i.e., the product of the factor-level-combination of the factors funniness and valence). However, the calculated 2 (funniness: funny/not funny) × 2 (valence: positive/negative) between-subject ANOVA on *purchase intention* showed neither significant main effects [funniness or arousal: *F*(1, 407) = 0.13, *p* = 0.722; valence: *F*(1, 407) = 0.39, *p* = 0.534], nor a significant Funniness × Valence interaction, *F*(1, 407) = 0.07, *p* = 0.786. Hence, how participants *subjectively* perceive the joke (e.g., positive, or negative) does not affect their purchase intention.^5^ What is the effect of the content of the joke (i.e., joke type) on purchase intent?

### 3.3 Position and joke type

Based on the literature, we assume that the position or joke type has a different influence on purchase intention depending on whether the posted healthy food product was recognized or not (i.e., the participant viewed it with attention or without). We therefore conducted 2 separate analyses in this regard to reflect this distinction in terms of attention.

#### 3.3.1 Healthy food product recognized

**Figure 3:**
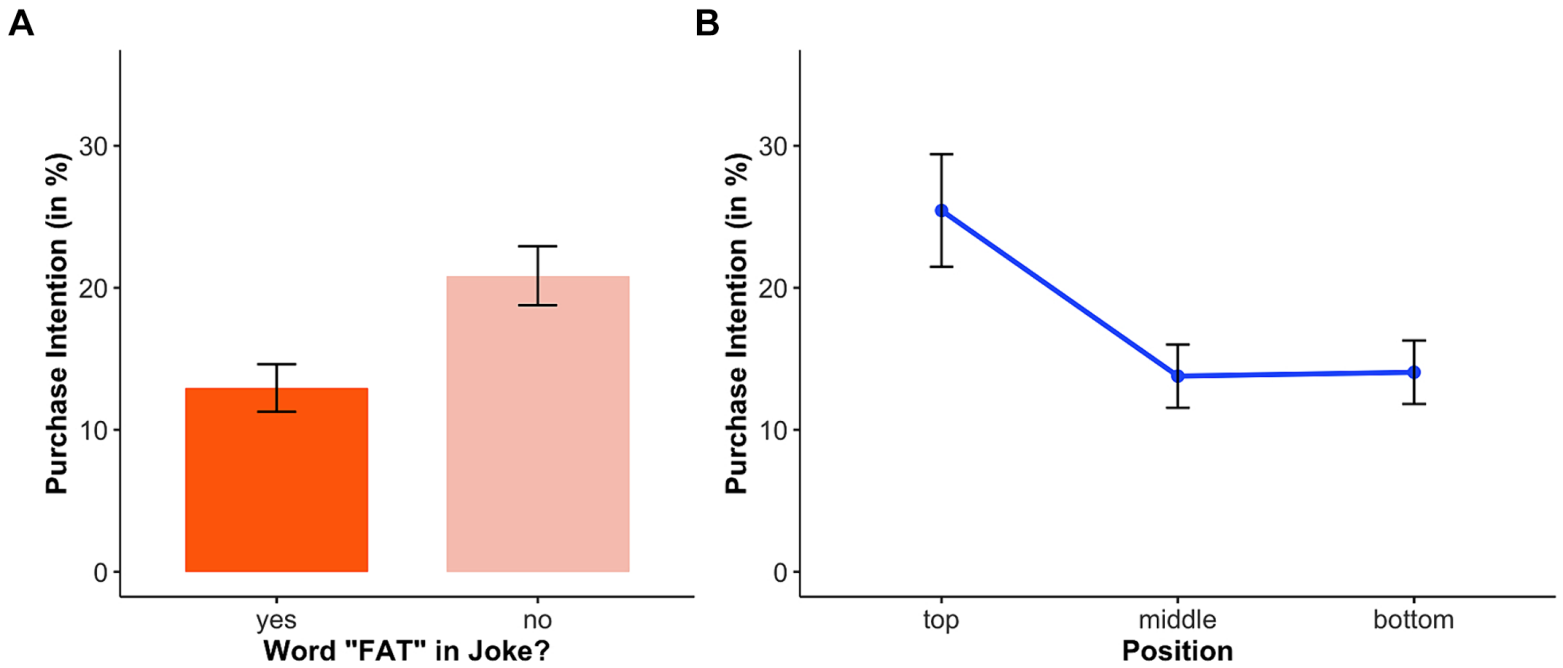
Purchase intention in relation to joke content **(A)** and position **(B)**.

A calculated 1-factorial (position: top, middle, bottom) ANOVA showed that the joke/healthy food product *position* had no significant effect on purchase intention, *F*(2, 214) = 0.47, *p* = 0.623. However, a calculated 1-factorial (joke type: Google, career, shape, and elevator) ANOVA showed that the joke *type* had a significant effect on purchase intention, *F*(3, 213) = 3.37, *p* < 0.05. Thereby, a planned contrast (between the jokes that contain the word fat in the joke and those that do not) showed that the two jokes containing the word fat had a smaller influence than the other two jokes, *F*(1, 213) = 8.24, *p* < 0.01 (see Figure 3). This suggests that participants’ intention to buy a healthy food item decreases as soon as the word “fat” appears in the joke.

#### 3.3.2 Healthy food product NOT recognized

Here a calculated 1-factorial (position: top, middle, bottom) ANOVA showed that the joke/healthy food product *position* had a significant effect—unlike above—on purchase intention, *F*(2, 191) = 5.26, *p* < 0.01 (see Figure 3). We found that the top position had a greater (positive) influence on purchase intention than the middle or bottom positions (top^6^ vs. middle, respectively top vs. bottom: *p* < 0.05, middle vs. bottom: *p* > 0.99). However, a calculated 1-factorial (joke type: Google, career, shape, and elevator) ANOVA showed that the joke type had no significant effect on purchase intention, *F*(3, 190) = 1.72, *p* = 0.165; which makes sense, since their contents were probably not processed.

## 4 Discussion

The aim of the study was to find out whether or not humorous messages in advertisements of healthy foods on social media platforms have an impact on people’s purchasing behavior, and if so, whether these humorous messages could be used to combat obesity. We found that the joke type or joke content had an effect if the advertised product was recognized; the presence of the word “fat” thereby had a negative effect. On the other hand, if the product was not recognized, only the positioning of the joke/product played a role, with the top position being the most effective. Given that about half of the participants (47%) did not recognize the product, a more promising strategy than using jokes in the context of social media would be to place the product of interest at the very top of the social media feed. This would take advantage of the so-called “nudging” strategy (see introduction), which assumes that by making small changes in the choice architecture (e.g., putting the Coke in the refrigerator behind the milk), you can *predictably* steer people’s decisions in a particular direction that is more beneficial to them (e.g., eating healthier; see Thaler and Sunstein, 2021). Both product position and the use of humor (especially the latter) can be classified as so-called *commercial nudges*, since they benefit both the retailer and the consumer (see also Congiu and Moscati, 2022), provided that the advertised product is healthy. However, the benefit to the consumer (in this case a healthier diet) does not exempt us from addressing the debate on the ethical and moral implications of the use of jokes (or humor) in advertising (see analogous discussion on the nudging approach and libertarian paternalism, respectively: Lembcke et al., 2019; Sunstein, 2019). This debate is especially relevant given Beard’s (2008) observation, that offensive themes are quite prevalent in U.S. advertisements and Förster and Brantner’s (2016) observation that ethics-violating advertisements are perceived as less unethical if they contain humor. We always need to remain aware of the fact that using words such as “fat” in jokes might *offend* audiences. One question that needs to be answered in the future, therefore, is: can we use humor, and if so, how can we use it in a non-offensive way? Likewise, we must not forget that humor in healthcare contexts has long been shown to have a positive impact on people’s well-being,^7^ for example by helping them to cope with stressful situations (see Martin and Lefcourt, 1983; Savage et al., 2017 for a review).

Beside this ethical-moral debate, we found that the intention to buy the healthy food product was quite low, around 17%. Hence, the healthy “low carb muesli” did not seem to be a very popular product. This raises the question whether we would have observed differential effects in the presence of a more *popular* product (see, for example, Dahlén and Lange, 2005, for differential effects).

Furthermore, the absence of any effects (on liking of the healthy food post and purchase intention) of funniness (kind of arousal) or valence (or both) evoked by the joke requires further investigation. Although the jokes led to the ads being perceived as funny, the way the humor was perceived seemed to be of a fragile nature, such that, for example, the “funny-ness” had no influence on purchase intentions, either because the “funny-ness” was not strong enough or too short-lived. Accordingly, Yoon (2018; see also Aylesworth and MacKenzie, 1998) postulated that perceived humor (in the face of a humorous advertisement) must be further reinforced by placing the recipient in an *emotion-triggering* context (e.g., low arousal/positive valence) beforehand, for example, by means of a movie. Accordingly, Yoon (2018) showed that valence and arousal manipulations influence *perceived humor* (with perceived arousal of the ad as a mediator). However, a closer look shows that a negative effect between arousal and perceived humor was found only when valence was positive (i.e., the lower the arousal, the funnier the ad was perceived to be). This result is consistent with the theoretical assumption that the greater the surprise, the greater the perceived humor (generated here by the incongruence of low arousal state and humor; Alden et al., 2000; Woltman Elpers et al., 2004); provided that the incongruence can be successfully resolved (understanding the punch line). However, he also found no direct effect of perceived humor on purchase intention, only an indirect one (via attitude towards the ad and then attitude towards the brand; see also Aylesworth and MacKenzie, 1998, for confirmation of the correlation between, for example, perceived humor and attitude towards the ad).

The absence of the effect (i.e., influence of, for example, valence on purchase intention) in our study is therefore not unusual. Accordingly, our study shows that it is not the *feelings* or emotions evoked by the joke that are decisive, but rather its *content*. In this regard, it might be worth paying more attention to the humor-message relationship (e.g., semantic, syntactic). For example, Cline and Kellaris (2007) found that when the description of an attribute (e.g., “delicate, earthy flavor”) of a product (e.g., coffee) is semantically related to the joke (words are part of the joke), participants remember the description better. Hence, it might be worth studying humor-message relationships regarding purchase intention.

In addition to using a broader range of products (e.g., in terms of their popularity) and exploring more intelligent humor (that is, humor that relates to the nature and function of products), another study limitation could be that we did not collect (for ethical reasons) participants’ body mass index (BMI). The BMI could have provided us with further meaningful insights into how the participants’ weight affected their perception of humor and, accordingly, their purchase intention.

Nonetheless, this has been one of the first studies, if not the first study, which attempts to examine the effects of humor—embedded in social media feeds—on healthy food purchases as a possible solution to the obesity crisis. Although humor seems to work in some ways, future studies need to examine the “when and how” in more detail, without the fear of appearing frivolous.

## Data availability statement

The datasets presented in this article are not readily available because only aggregated data is available by request. Requests to access the datasets should be directed to ER, reij@zhaw.ch.

## Ethics statement

The studies involving humans were approved by Kanton Zürich, Kantonale Ethikkommission. The studies were conducted in accordance with the local legislation and institutional requirements. The participants provided their written informed consent to participate in this study.

## Author contributions

ER: Conceptualization, Formal analysis, Methodology, Writing – original draft, Writing – review & editing. LLV: Conceptualization, Formal analysis, Methodology, Writing – original draft, Writing – review & editing. DC: Conceptualization, Formal analysis, Methodology, Writing – original draft, Writing – review & editing. JZ: Conceptualization, Software, Writing – review & editing. SB-R: Conceptualization, Software, Writing – review & editing. LB: Writing – original draft.

## Footnotes

^1^See Moran (1996), who notes that the “fear of appearing frivolous” (p. 32) might be one reason for the lack of research on applied humor.

^2^Note that the two jokes containing the word “fat” were the joke shown in Figure 1 (in English: 2020 in brief: [and then in the Google search bar] “how to get” with the suggested answers “how to get COVID”, and “how to lose belly fat”) and in plain text “Du kannst alles werden”, haben sie gesagt. Wieso muss ich mich ausgerechnet für “fett” entscheiden? (“You can become anything you want”, they said. Why did I choose “fat” of all things?). The other two jokes without the word “fat” and also in plain text were: “Ich weiss gar nicht, was alle haben, ich bin top in Form. Rund ist eine Form” (“I do not see the problem, I’m in great shape. After all, round is a shape”) and “Wenn beim Einsteigen in den Lift alle auf die Anzeige fürs zugelassene Maximalgewicht schauen …” (“You know it’s bad if they stare at the weight limit sign when you enter the elevator …”; see So et al., 2016).

^3^It should be noted here that participants were given the following notice during instructions: “This survey may contain discriminatory statements. These are intended to answer a scientific question and do not reflect the position of the Applied Cognitive Psychology team”. So, the participants could—if they wanted to—stop at this point.

^4^Statistics were only calculated over the full “not moved (= 0 incl.)” set (*N* = 411).

^5^The same (non-significant) pattern of results is also found regarding the liking of the healthy food post. This not only counts for calculation using an ANOVA (all *F*’s < = 0.068; *p*’s > 0.794), but also using point-biserial correlations (all *r*’s < = 0.008; all *p*’s > 0.866); only the funniness and valence appear to correlate with each other (*r* = 0.439, *p* < 0.001). Hence, funniness and valence also had no spill-over effect on liking of the healthy food post.

^6^All values are Bonferroni corrected.

^7^See, for example, Jiang et al. (2019) for quantitative but not qualitative differences between Western and Eastern cultures in the relationship between humor and well-being.

## References

[ref1] Alden D. L. Mukherjee A. Hoyer W. D. (2000). Extending a contrast resolution model of humor in television advertising: the role of surprise. Humor 13, 193–217. doi: 10.1515/humr.2000.13.2.193

[ref2] Auty S. Lewis C. (2004). Exploring children's choice: the reminder effect of product placement. Psychol. Mark. 21, 697–713. doi: 10.1002/mar.20025

[ref3] Aylesworth A. B. Mackenzie S. B. (1998). Context is key: the effect of program-induced mood on thoughts about the ad. J. Advert. 27, 17–31. doi: 10.1080/00913367.1998.10673550

[ref4] Bartos R. Dunn T. F. (1976). Advertising and consumers: New perspectives. New York: American Association of Advertising Agencies.

[ref5] Beard F. K. (2008). Advertising and audience offense: the role of intentional humor. J. Mark. Commun. 14, 1–17. doi: 10.1080/13527260701467760

[ref6] Boyland E. J. Nolan S. Kelly B. Tudur-Smith C. Jones A. Halford J. C.. (2016). Advertising as a cue to consume: a systematic review and meta-analysis of the effects of acute exposure to unhealthy food and nonalcoholic beverage advertising on intake in children and adults. Am. J. Clin. Nutr. 103, 519–533. doi: 10.3945/ajcn.115.120022, PMID: 26791177

[ref7] Bragg M. Lutfeali S. Greene T. Osterman J. Dalton M. (2021). How food marketing on Instagram shapes adolescents’ food preferences: online randomized trial. J. Med. Internet Res. 23:e28689. doi: 10.2196/28689, PMID: 34677136 PMC8571690

[ref8] Cadwell F. (1981). Funny makes money: Cadwell Davis Savage Advertising.

[ref9] Chung C.-F. Agapie E. Schroeder J. Mishra S. Fogarty J. Munson S. A. (2017). When personal tracking becomes social: examining the use of Instagram for healthy eating. Proc. SIGCHI Conf. Hum. Factor Comput. Syst. 2017, 1674–1687. doi: 10.1145/3025453.3025747, PMID: 28516174 PMC5432132

[ref10] Cline T. W. Kellaris J. J. (2007). The influence of humor strength and humor—message relatedness on ad memorability: a dual process model. J. Advert. 36, 55–67. doi: 10.2753/joa0091-3367360104

[ref11] Cohen M. A. Cavanagh P. Chun M. M. Nakayama K. (2012). The attentional requirements of consciousness. Trends Cogn. Sci. 16, 411–417. doi: 10.1016/j.tics.2012.06.013 22795561

[ref12] Congiu L. Moscati I. (2022). A review of nudges: definitions, justifications, effectiveness. J. Econ. Surv. 36, 188–213. doi: 10.1111/joes.12453

[ref13] Dahlén M. Lange F. (2005). Advertising weak and strong brands: who gains? Psychol. Mark. 22, 473–488. doi: 10.1002/mar.20069

[ref14] Dunlop S. Freeman B. Jones S. C. (2016). Marketing to youth in the digital age: the promotion of unhealthy products and health promoting behaviours on social media. Media Commun. 4, 35–49. doi: 10.17645/mac.v4i3.522

[ref15] Eisend M. (2009). A meta-analysis of humor in advertising. J. Acad. Mark. Sci. 37, 191–203. doi: 10.1007/s11747-008-0096-y

[ref16] Förster K. Brantner C. (2016). Masking the offense? An ethical view on humor in advertising. *J. Media* Ethics 31, 146–161. doi: 10.1080/23736992.2016.1188013

[ref17] Freeman B. Kelly B. Baur L. Chapman K. Chapman S. Gill T.. (2014). Digital junk: food and beverage marketing on Facebook. Am. J. Public Health 104, e56–e64. doi: 10.2105/AJPH.2014.302167, PMID: 25322294 PMC4232106

[ref18] Gelb B. D. Pickett C. M. (1983). Attitude-toward-the-ad: links to humor and to advertising effectiveness. J. Advert. 12, 34–42. doi: 10.1080/00913367.1983.10672838

[ref19] Goodrich K. (2011). Anarchy of effects? Exploring attention to online advertising and multiple outcomes. Psychol. Mark. 28, 417–440. doi: 10.1002/mar.20371

[ref20] Grill-Spector K. Kanwisher N. (2005). Visual recognition: as soon as you know it is there, you know what it is. Psychol. Sci. 16, 152–160. doi: 10.1111/j.0956-7976.2005.00796.x, PMID: 15686582

[ref21] Hingle M. Kunkel D. (2012). Childhood obesity and the media. Pediatr. Clin. N. Am. 59, 677–692. doi: 10.1016/j.pcl.2012.03.021 22643173

[ref22] Jaacks L. M. Vandevijvere S. Pan A. McGowan C. J. Wallace C. Imamura F.. (2019). The obesity transition: stages of the global epidemic. Lancet 7, 231–240. doi: 10.1016/S2213-8587(19)30026-9, PMID: 30704950 PMC7360432

[ref23] Jiang T. Li H. Hou Y. (2019). Cultural differences in humor perception, usage, and implications. Front. Psychol. 10:123. doi: 10.3389/fpsyg.2019.00123, PMID: 30761053 PMC6361813

[ref24] Lembcke T.-B. Engelbrecht N. Brendel A. B. Kolbe L. M. (2019). To nudge or not to nudge: ethical considerations of digital nudging based on its behavioral economics roots. In: ECIS. 27th European conference on information systems, 2019, Stockholm-Uppsala, Sweden

[ref25] Ling J. Chen S. Zahry N. R. Kao T.-S. A. (2023). Economic burden of childhood overweight and obesity: a systematic review and meta-analysis. Obes. Rev. 24:e13535. doi: 10.1111/obr.13535, PMID: 36437105 PMC10078467

[ref26] Madden T. J. Weinberger M. G. (1984). Humor in advertising: a practitioner view. J. Ad. Res. 24, 23–29.

[ref27] Martin R. A. Lefcourt H. M. (1983). Sense of humor as a moderator of the relation between stressors and moods. J. Pers. Soc. Psychol. 45, 1313–1324. doi: 10.1037/0022-3514.45.6.1313

[ref28] Mohajan D. Mohajan H. K. (2023). Obesity and its related diseases: a new escalating alarming in global health. J. Inno. Med. Res. 2, 12–23. doi: 10.56397/jimr/2023.03.04

[ref29] Moran C. C. (1996). Short-term mood change, perceived funniness, and the effect of humor stimuli. Behav. Med. 22, 32–38. doi: 10.1080/08964289.1996.9933763, PMID: 8805959

[ref30] Nguyen T. A. Coursaris C. K. Léger P. M. Sénécal S. Fredette M. (2020). Effectiveness of banner ads: an eye tracking and facial expression analysis. In: HCI in business, government and organizations. 7th International Conference, HCIBGO 2020. Held as part of the 22nd HCI International Conference, HCII 2020, Copenhagen, Denmark, July19–24, 2020, Proceedings, Vol. 12204, pp. 445–455. Springer, doi: 10.1007/978-3-030-50341-3_34

[ref31] Okunogbe A. Nugent R. Spencer G. Ralston J. Wilding J. (2021). Economic impacts of overweight and obesity: current and future estimates for eight countries. BMJ Glob. Health 6:e006351. doi: 10.1136/bmjgh-2021-006351, PMID: 34737167 PMC8487190

[ref32] Petty R. E. Cacioppo J. T. Schumann D. (1983). Central and peripheral routes to advertising effectiveness: the moderating role of involvement. J. Consum. Res. 10, 135–146. doi: 10.1086/208954

[ref33] Piacentini M. MacFadyen L. Eadie D. (2000). Corporate social responsibility in food retailing. Int. J. Retail Distrib. Manag. 28, 459–469. doi: 10.1108/09590550010356822

[ref34] Potvin Kent M. Pauzé E. Roy E.-A. de Billy N. Czoli C. (2019). Children and adolescents’ exposure to food and beverage marketing in social media apps. Pediatr. Obes. 14:e12508. doi: 10.1111/ijpo.12508, PMID: 30690924 PMC6590224

[ref35] Reagan R. Filice S. Santarossa S. Woodruff S. J. (2020). # ad on Instagram: investigating the promotion of food and beverage products. J. Soc. Media Soc. 9, 1–28.

[ref36] Reijnen E. Kühne S. J. von Gugelberg H. M. Crameri A. (2019). Nudged to a menu position: the role of “I’m loving it”! J. Consumer Policy 42, 441–453. doi: 10.1007/s10603-019-09413-4

[ref37] Reynolds J. P. Archer S. Pilling M. Kenny M. Hollands G. J. Marteau T. M. (2019). Public acceptability of nudging and taxing to reduce consumption of alcohol, tobacco, and food: a population-based survey experiment. Soc. Sci. Med. 236:112395. doi: 10.1016/j.socscimed.2019.112395, PMID: 31326778 PMC6695289

[ref38] Savage B. M. Lujan H. L. Thipparthi R. R. DiCarlo S. E. (2017). Humor, laughter, learning, and health! A brief review. Adv. Physiol. Educ. 41, 341–347. doi: 10.1152/advan.00030.2017, PMID: 28679569

[ref39] So J. Prestin A. Lee L. Wang Y. Yen J. Chou W.-Y. S. (2016). What do people like to “share” about obesity? A content analysis of frequent retweets about obesity on twitter. Health Commun. 31, 193–206. doi: 10.1080/10410236.2014.940675 26086083

[ref40] Sternthal B. Craig C. S. (1973). Humor in advertising. J. Mark. 37, 12–18. doi: 10.1177/002224297303700403

[ref41] Strick M. van Baaren R. B. Holland R. W. van Knippenberg A. (2009). Humor in advertisements enhances product liking by mere association. J. Exp. Psychol. Appl. 15, 35–45. doi: 10.1037/a0014812, PMID: 19309215

[ref42] Sturm R. Cohen D. A. (2009). Zoning for health? The year-old ban on new fast-food restaurants in South LA: the ordinance isn't a promising approach to attacking obesity. Health Aff. 28, w1088–w1097. doi: 10.1377/hlthaff.28.6.w1088, PMID: 19808703 PMC2866128

[ref43] Sunstein C. R. (2019). Nudging: a very short guide. Bus. Econ. 54, 127–129. doi: 10.1057/s11369-018-00104-5

[ref44] Sutherland M. (2008). Advertising and the mind of the consumer: What works, what Doesn’t, and why. 3rd Edn. Crows Nest, NSW, Australia: Allen & Unwin.

[ref45] Thaler R. H. Sunstein C. R. (2021). Nudge: the final edition. New York: Penguin.

[ref46] Thornton L. E. Cameron A. J. McNaughton S. A. Worsley A. Crawford D. A. (2012). The availability of snack food displays that may trigger impulse purchases in Melbourne supermarkets. BMC Public Health 12, 1–8. doi: 10.1186/1471-2458-12-194, PMID: 22420759 PMC3386861

[ref47] Turnwald B. P. Anderson K. G. Markus H. R. Crum A. J. (2022). Nutritional analysis of foods and beverages posted in social media accounts of highly followed celebrities. JAMA Netw. Open 5:e2143087. doi: 10.1001/jamanetworkopen.2021.43087, PMID: 35019982 PMC8756336

[ref48] Wansink B. Hanks A. S. (2013). Slim by design: serving healthy foods first in buffet lines improves overall meal selection. PLoS One 8:e77055. doi: 10.1371/journal.pone.0077055, PMID: 24194859 PMC3806736

[ref49] Weinberger M. G. Gulas C. S. (1992). The impact of humor in advertising: a review. J. Advert. 21, 35–59. doi: 10.1080/00913367.1992.10673384

[ref50] Woltman Elpers J. L. C. M. Mukherjee A. Hoyer W. D. (2004). Humor in television advertising: a moment-to-moment analysis. J. Consum. Res. 31, 592–598. doi: 10.1086/425094

[ref51] World Health Organization. (2020). Obesity and overweight. Available at: https://www.who.int/news-Room/fact-Sheets/detail/obesity-And-Overweigh (Accessed April 15, 2024).

[ref52] Yoon H. J. (2018). Creating the mood for humor: arousal level priming in humor advertising. J. Consum. Mark. 35, 491–501. doi: 10.1108/jcm-01-2017-2074

